# Platelets mediate inflammatory monocyte activation by SARS-CoV-2 spike protein

**DOI:** 10.1172/JCI150101

**Published:** 2022-02-15

**Authors:** Tianyang Li, Yang Yang, Yongqi Li, Zhengmin Wang, Faxiang Ma, Runqi Luo, Xiaoming Xu, Guo Zhou, Jianhua Wang, Junqi Niu, Guoyue Lv, Ian N. Crispe, Zhengkun Tu

**Affiliations:** 1 Institute of Translational Medicine, The First Hospital of Jilin University, Changchun, Jilin, China.; 2 Department of Infectious Disease, The Second Affiliated Hospital of Guangzhou Medical University, Guangzhou, Guangdong, China.; 3 Guangzhou Institutes of Biomedicine and Health, Chinese Academy of Sciences, Guangzhou, Guangdong, China.; 4 Institute of Liver Diseases, The First Hospital of Jilin University, Changchun, Jilin, China.; 5 Department of Laboratory Medicine and Pathology, University of Washington, Seattle, Washington, USA.

**Keywords:** COVID-19, Inflammation, Monocytes, Platelets

## Abstract

Infection with SARS-CoV-2, the causative agent of COVID-19, causes mild to moderate disease in most patients but carries a risk of morbidity and mortality. Seriously affected individuals manifest disorders of hemostasis and a cytokine storm, but it is not understood how these manifestations of severe COVID-19 are linked. Here, we showed that the SARS-CoV-2 spike protein engaged the CD42b receptor to activate platelets via 2 distinct signaling pathways and promoted platelet-monocyte communication through the engagement of P selectin/PGSL-1 and CD40L/CD40, which led to proinflammatory cytokine production by monocytes. These results explain why hypercoagulation, monocyte activation, and a cytokine storm are correlated in patients severely affected by COVID-19 and suggest a potential target for therapeutic intervention.

## Introduction

A major pandemic is in progress due to SARS-CoV-2, a virus with 79% sequence homology with SARS-CoV and 50% homology with MERS-CoV (1). It is less pathogenic than either of these infections but has spread much more widely. Despite promising vaccine efforts, COVID-19 continues to cause severe morbidity and mortality in many patients. While the best-known manifestation of severe COVID-19 is pneumonia leading to respiratory distress, severe cases also feature systemic inflammation (including a “cytokine storm” that contributes to disease severity in many virus infections) and hypercoagulation (2). Multiple clinical studies have shown that platelets are involved in COVID-19 (3–5). Thus, platelet counts were in the lower range in patients with severe or non-severe COVID-19 in comparison to healthy volunteers (5), and thrombocytopenia was correlated with a poor outcome (3). Platelets express TLRs and actively bind to microorganisms via pattern recognition receptors (6), which may elicit platelet activation and eventually favor the occurrence of thrombosis-related cardiovascular events (7).

Aberrant coagulation is frequent in severe and critically ill patients with COVID-19 (8). Dysregulation of the innate immune response also contributes to the cytokine storm seen in severe, life-threatening SARS-CoV-2 infection (9). Activation of coagulation pathways during the immune response to infection results in overproduction of proinflammatory cytokines, leading to multiorgan injury (10, 11). Platelets have the potential to interact with circulating monocytes, and abundant inflammatory monocytes were found in the bronchioalveolar lavage fluid from patients with severe COVID-19 pneumonia (12). Such inflammatory monocytes are a major source of the proinflammatory cytokines that constitute the cytokine storm (12, 13).

SARS-CoV-2 gains access to many cell types by binding of the viral spike protein to the host cell angiotensin converting enzyme-2 (ACE-2), but alternative pathways of infection have been described (14). We were therefore interested in determining how SARS-CoV-2 engages with platelets, and we found a unique receptor, the CD42b molecule. The clinical association of coagulopathy and hyperinflammation with severe disease encouraged us to test whether platelets under the influence of SARS-CoV-2 might activate monocytes. We found that such cross-talk indeed occurred and identified the molecules involved, CD40L binding to CD40 and P selectin binding to P selectin glycoprotein ligand 1 (PSGL-1). This mechanism depends on the SARS-CoV-2 spike protein but appears to be fully independent of the ACE-2 infection pathway that is important in other cells.

## Results

### SARS-CoV-2 induced platelet activation by spike protein.

**Figure 1:**
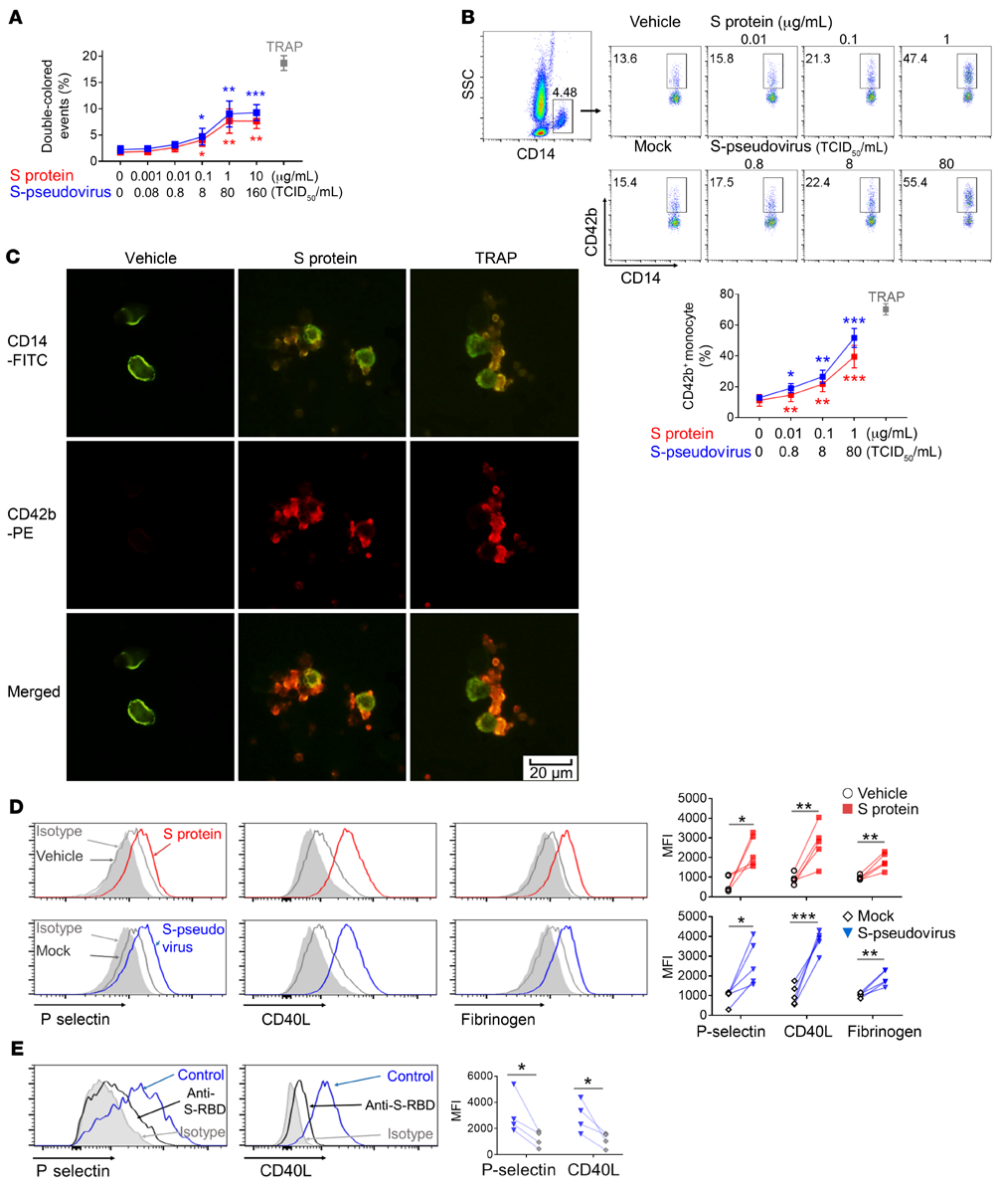
SARS-CoV-2–induced platelet-monocyte aggregation and P selectin/CD40L expression on platelets by spike protein. (**A**) PKH26- or CFSE-labeled platelets from healthy donors (*n =* 5) were mixed at 1:1 and incubated with increasing concentrations of spike protein or S-pseudovirus, and aggregates were detected by flow cytometry. Double-colored events indicated the platelet aggregation, and TRAP was used as the positive control. (**B**) Peripheral blood from healthy donors (*n =* 5) was stimulated by spike protein or S-pseudovirus at indicated concentration and analyzed by flow cytometry. Platelet-monocyte aggregation was evaluated using the percentage of CD42b^+^/CD14^+^ cells. (**C**) Platelet-monocyte aggregation was visualized by fluorescence microscopy; scale bar: 20 μm. (**D**) Purified platelets from healthy donors (*n =* 5) were incubated with spike protein or S-pseudovirus. P selectin, CD40L expression, and fibrinogen binding were shown by histogram and MFI. (**E**) Purified platelets (*n =* 4) were pretreated with isotype antibody (indicated as control) or anti-spike RBD before incubation with S-pseudovirus; P selectin and CD40L expression are shown. Mean with SD and *P* value by paired Student’s *t* test are displayed. *P < 0.05; **P < 0.01; ***P < 0.001.

To test whether SARS-CoV-2 induces platelet activation directly, we first purified platelets from healthy donors (Supplemental Figure 1; supplemental material available online with this article; https://doi.org/10.1172/JCI150101DS1), and a 50/50 mixture of PKH26- and CFSE-labeled platelets was cocultured with either recombinant SARS-CoV-2 spike protein or a vesicular stomatitis virus (VSV) pseudotyped virus expressing spike (termed S-pseudovirus); TRAP (thrombin receptor activating peptide, 20 μM) was the positive control. The platelet aggregation was assayed by flow cytometry based on double-colored events. The result showed that both spike protein (0.001–1 μg/mL) and S-pseudovirus (0.08–80 tissue culture ID_50_/mL [TCID_50_/mL]) induced platelet aggregation in a dose-dependent manner. The effect was saturating at 160 TCID_50_/mL of S-pseudovirus and 10 μg/mL of spike protein (Figure 1A and Supplemental Figure 2A). Using RGD (Arg-Gly-Asp) peptide as an αIIbβ3 inhibitor, we observed that RGD peptide blocked spike protein– or S-pseudovirus–induced platelet aggregation (Supplemental Figure 2B). In addition, using platelet aggregometry in platelet-rich plasma under stirring conditions, we further confirmed that both spike protein and S-pseudovirus induced platelet aggregation (Supplemental Figure 2C).

Activated platelets preferentially bind to monocytes and form platelet-monocyte aggregates, a more sensitive and robust marker of platelet activation than the expression of P selectin and CD40L (15, 16). We exposed whole blood from healthy donors to spike protein or S-pseudovirus, again using TRAP as a positive control. Platelet-monocyte aggregates were evaluated by observing that CD14^+^ monocytes adhered to CD42b^+^ platelets. We observed that both spike protein and S-pseudovirus caused a dose-dependent increase of platelet-monocyte aggregates (Figure 1B). The optimal concentrations to activate platelets were 80 TCID_50_/mL of S-pseudovirus and 1.0 μg/mL of spike protein and were used as the standard concentrations in subsequent experiments. We further confirmed that spike protein induced platelet-monocyte aggregates using microscopy (Figure 1C). In addition, purified platelets from healthy donors exposed to either spike protein or the S-pseudovirus caused an increase in P selectin, CD40L, and fibrinogen binding (Figure 1D). Moreover, the increases in both P selectin and CD40L were inhibited by antibody against the spike receptor-binding domain (S-RBD; Figure 1E). Therefore, we identified spike protein–induced platelet activation that could explain the aggregation of platelets with monocytes.

### SARS-CoV-2 activates platelets by spike protein binding with CD42b.

**Figure 2:**
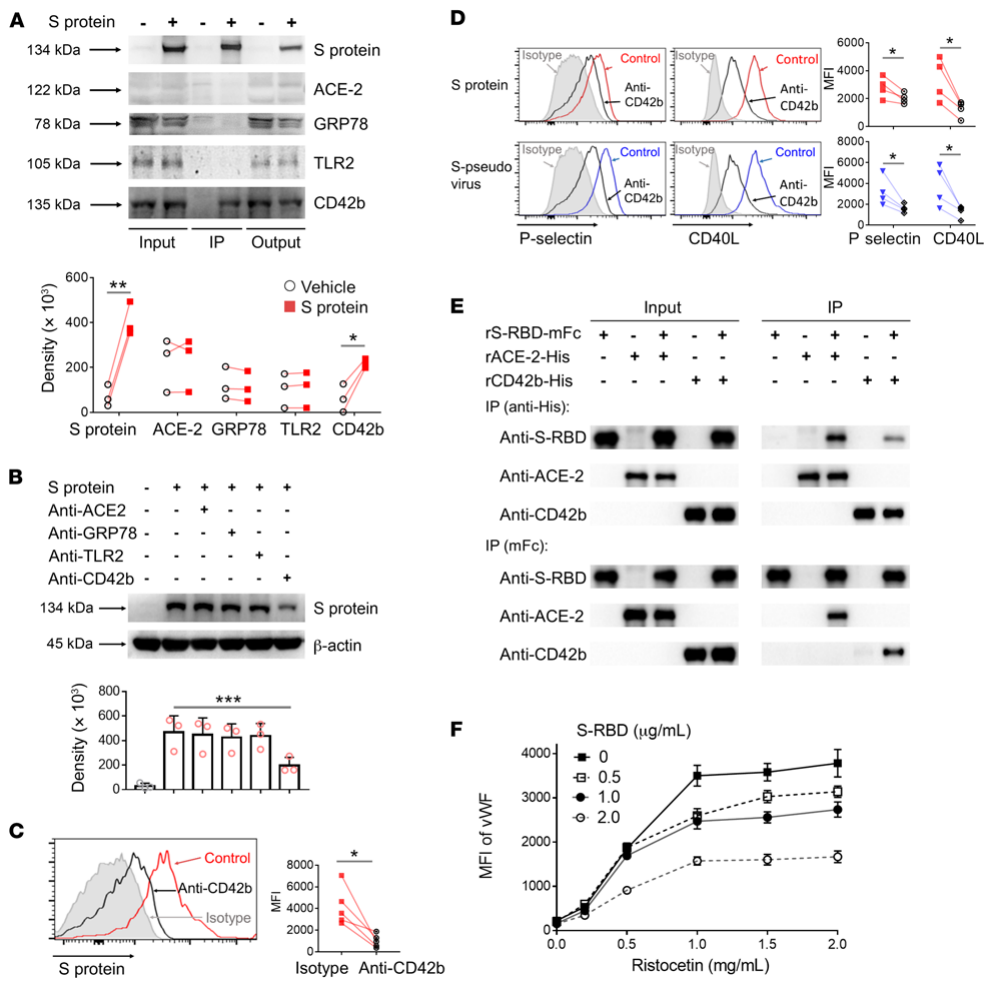
SARS-CoV-2 spike protein engaged with CD42b to induce P selectin/CD40L expression on platelets. (**A**) Purified platelets from healthy donors (*n =* 3) were incubated with or without spike protein, and Western blot analysis was performed using indicated antibody after IP for spike protein (IP lane). Density of IP lane was analyzed. (**B**) Purified platelets (*n =* 3) were pretreated with indicated antibody before incubation with spike protein; spike protein on platelets detected by Western blot. (**C** and **D**) Purified platelets were pretreated with anti-CD42b or isotype antibody before incubation by spike protein or S-pseudovirus; spike protein binding (*n =* 5) and the expressions of P selectin and CD40L (*n =* 4) on platelets were examined by flow cytometry. As anti-CD42b control, red controls were indicated as spike protein and isotype antibody; blue controls indicated as S-pseudovirus and isotype antibody. (**E**) Recombinant S-RBD was incubated with ACE-2 or CD42b, and their interaction was measured by co-IP. (**F**) Fixed platelets were incubated with S-RBD, and platelet-vWF complexes were detected by flow cytometry using recombinant vWF and ristocetin. Comparisons were made with paired Student’s *t* test, except in **B**, which was assessed by 1-way ANOVA with Dunnett’s multiple-comparison test. Mean with SD and *P* value are displayed. *P < 0.05; **P < 0.01; ***P < 0.001.

ACE-2 (17), GRP78 (18), TLR2 (19), and CD42b (20) are all potential receptors for SARS-CoV-2 on platelets. To identify the receptor that spike protein binds on platelets, we treated platelets from healthy donors with recombinant spike; conducted IP using anti-spike antibody; and estimated the concentrations of the SARS-CoV-2 receptors ACE-2, GRP78, TLR2, and CD42b by Western blotting. In 3 experiments, densitometry showed that only CD42b was increased by exposure to spike protein (Figure 2A). Conversely, antibodies against CD42b but not antibodies against ACE-2, GRP78, or TLR2 were able to inhibit binding of spike protein to platelets, as shown by Western blotting (Figure 2B). The effect was confirmed using flow cytometry to directly visualize spike protein on platelets (Figure 2C). Most strikingly, anti-CD42b antibody inhibited the upregulation of both P selectin and CD40L, induced by both spike protein and the S-pseudovirus (Figure 2D).

To further confirm that spike protein binds with CD42b, we performed an experiment for direct binding studies of recombinant spike protein to recombinant CD42b and ACE-2 using co-IP, reverse co-IP, and biolayer interferometry analysis. The results showed that spike protein bound to both CD42b and ACE-2, and the binding affinity of spike protein with CD42b was lower than ACE-2 (Figure 2E and Supplemental Figure 3). Our results also clearly showed that ACE-2 was not expressed on platelets, suggesting that ACE-2 was not involved in SARS-CoV-2 spike protein–induced platelet activation (Supplemental Figure 4).

To test whether spike protein blocks vWF binding to CD42b and interferes with platelet adherence to vWF, we also performed a vWF-binding assay, in which platelets were incubated with ristocetin and labeled vWF in the presence or absence of spike protein, to examine the formation of platelet-vWF complex. As shown in Figure 2F and Supplemental Figure 5A, S-RBD inhibited ristocetin-induced vWF binding on platelets, suggesting that spike binding with CD42b induced platelet aggregation, initiating a prothrombotic process in the absence of collagen. However, spike protein inhibited vWF-induced platelet agglutination in the presence of collagen (Supplemental Figure 5B). Spike protein binding with CD42b-induced platelet activation was not associated with collagen binding with glycoprotein VI–induced (GPVI-induced) platelet activation (Supplemental Figure 7).

From these data, we conclude that CD42b is the receptor through which spike protein both binds to platelets and activates their increased expression of ligands with the potential to bind monocytes.

### Signaling pathways within platelets.

**Figure 3:**
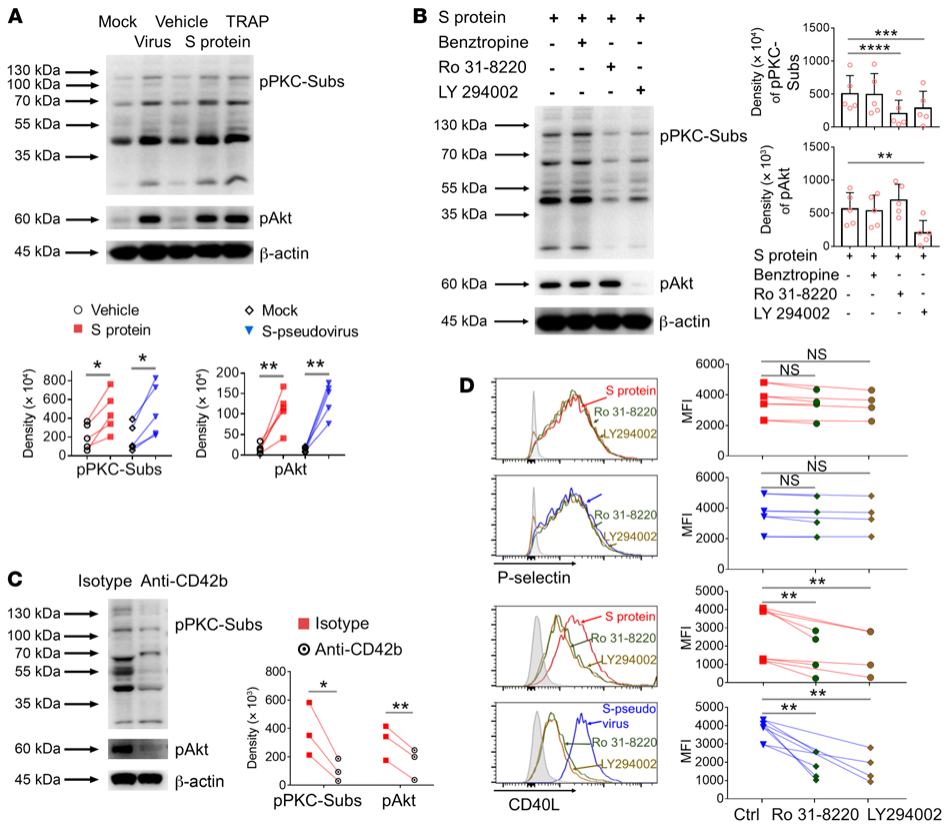
SARS-CoV-2 spike protein activated platelets via 2 distinct signaling pathways. (**A**) Purified platelets from healthy donors (*n =* 5) were incubated with spike protein or S-pseudovirus. Phosphorylation of PKC-substrates (pPKC-Subs, assessed PKC activation through the resulting phosphorylation of PKC substrates on specific serine residues) and Akt (pAkt) were detected by Western blot. TRAP was used as the positive control. (**B**) Purified platelets (*n =* 5) were pretreated with indicated inhibitors before incubation with spike protein. Phosphorylation of pPKC-Subs or Akt shown. (**C**) Purified platelets (*n =* 3) were pretreated with isotype or CD42b antibodies before incubation by spike protein. Phosphorylation of pPKC-Subs or Akt shown. (**D**) Purified platelets (*n =* 4) were pretreated with Ro31-8220 or LY294002 before incubation with spike protein or S-pseudovirus. P selectin and CD40L expression measured by flow cytometry. Comparisons between groups were measured by paired Student’s *t* test in **A** and **C**, or 1-way ANOVA with Dunnett’s multiple-comparison test in **B** and **D**. Mean with SD and *P* value are displayed. *P < 0.05; **P < 0.01; ***P < 0.001. **** *P* < 0.0001.

To test the involvement of platelet signaling pathways, we treated healthy donor platelets with spike protein or S-pseudovirus or TRAP as a control and used Western blotting to test for the phosphorylation of signaling intermediates. Both spike protein and S-pseudovirus caused the phosphorylation of PKC substrates and of AKT (Figure 3A). Platelets were pretreated with kinase inhibitors prior to spike protein or S-pseudovirus exposure. Benztropine, an inhibitor of G-protein–coupled receptors, had no effect. The PKC inhibitor Ro31-8220 inhibited the phosphorylation of PKC substrates as expected but increased the phosphorylation of AKT. The PI3K inhibitor LY294002 suppressed the phosphorylation of AKT and PKC substrates, suggesting it was upstream of PKC in the signaling cascade (Figure 3B). As might be expected, anti-CD42b antibody blocked the phosphorylation of PKC substrates and AKT (Figure 3C), confirming that these signaling events were also initiated by the spike-CD42b interaction.

The PI3K and AKT signaling pathways were not responsible for all the changes induced by spike protein and by S-pseudovirus in platelets. Thus, when the PI3K and PKC inhibitors were applied to platelets, the effects of both spike protein and S-pseudovirus on CD40L expression were completely neutralized, but the induction of P selectin expression was unaffected (Figure 3D). We conclude there are 2 distinct signaling cascades activated in platelets by the spike-CD42b interaction, but the pathway via AKT and PKC only acts on CD40L expression.

### Platelet-dependent spike protein–induced monocyte activation.

**Figure 4:**
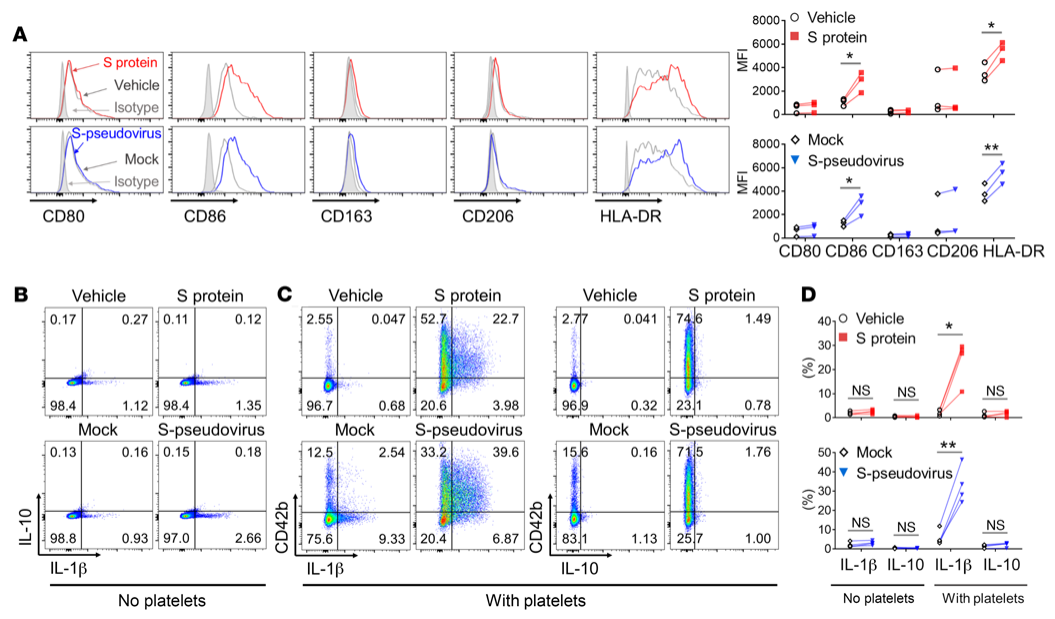
SARS-CoV-2–activated platelets induced monocyte differentiation toward a proinflammatory phenotype. Purified monocytes from healthy donors were cocultured with purified autologous platelets in the presence of spike protein or S-pseudovirus, and then washed to remove nonadherent surplus platelets. (**A**) After another 48 hours in culture, the cell surface expression of CD80, CD86, CD163, CD206, and HLA-DR on monocytes was analyzed by flow cytometry (*n =* 3). (**B**–**D**) The expression of IL-1β and IL-10 in monocytes was examined by intracellular cytokine staining after 12 hours in culture, and CD42b indicated platelet-monocyte adherence in cocultures (*n =* 4). *P* value by paired Student’s *t* test is displayed. *P < 0.05; **P < 0.01.

Monocytes are a major source of proinflammatory cytokines, and monocyte activation is a prominent feature of severe COVID-19 (12, 21). Therefore, we tested the extent to which SARS-CoV-2 spike protein– or S-pseudovirus–induced platelet activation resulted in proinflammatory monocyte activation. We found that 2 markers of proinflammatory monocyte activation, namely CD86 and HLA-DR, were reproducibly elevated by both spike protein and S-pseudovirus in the presence of platelets (Figure 4A).

When monocytes alone were exposed to either recombinant spike protein or to S-pseudovirus and LPS as a positive control, there was a minimal increase in IL-1β revealed by cytoplasmic staining (Figure 4B). Critically, when platelets were present, recombinant spike protein and S-pseudovirus strongly induced IL-1β staining in monocytes, and the IL-1β was mainly found in monocytes that were also stained for CD42b, indicating they were bound to platelets. (Figure 4C). The selective expression and production of IL-1β, and not IL-10, was strictly platelet-dependent in 4 independent experiments and statistically significant. There was a similar effect on IL-6 and TNF-α (Figure 4D and Supplemental Figure 8).

### Platelet-dependent spike protein–induced monocyte activation through the engagement of P selectin/PGSL-1 and CD40L/CD40.

**Figure 5:**
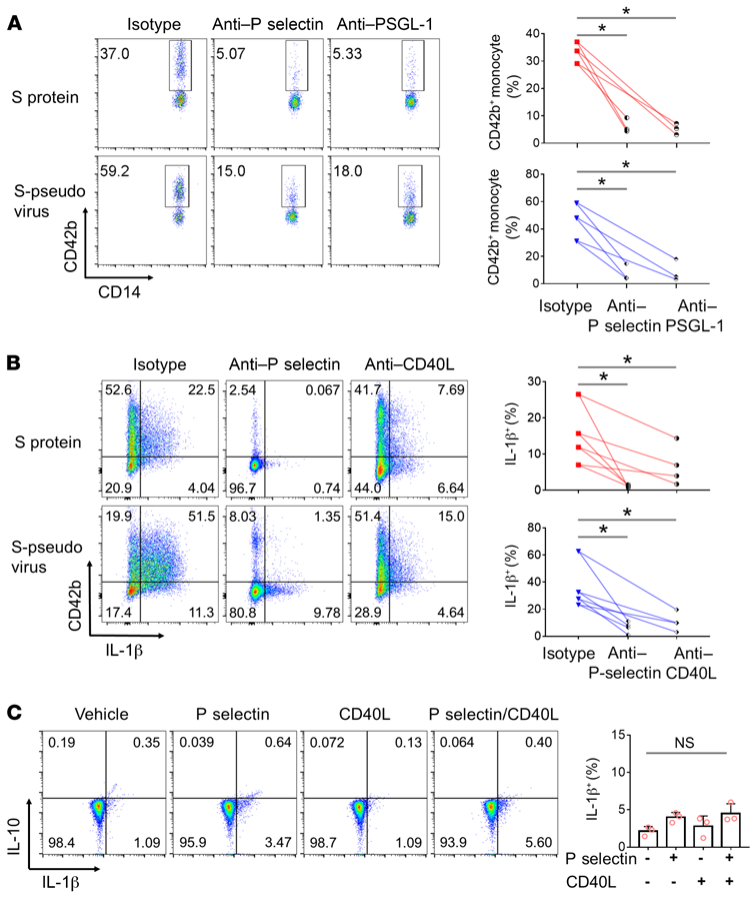
SARS-COV-2–activated platelets mediated inflammatory monocyte activation through the engagement of P selectin/PGSL-1 and CD40L/CD40. (**A**) Peripheral blood from healthy donors (*n =* 3) was stimulated by spike protein or S-pseudovirus in the presence of anti–P selectin or anti–PSGL-1. Platelet-monocyte aggregation was evaluated using the percentage of CD42b^+^/CD14^+^ cells. (**B**) Anti–P selectin or anti-CD40L was added into monocyte-platelet cocultures with spike protein or S-pseudovirus. IL-1β expression in monocytes (*n =* 4) was measured. (**C**) Purified monocytes (*n =* 3) were cultured with/without recombinant P selectin and/or CD40L, and their expression of IL-1β and IL-10 is shown. Mean with SD and *P* value by paired Student’s *t* test are displayed. *P < 0.05.

To further investigate the mechanism by which spike protein–activated platelets mediated inflammatory monocyte activation, we tested the effect of antibodies against both P selectin and its ligand, PSGL-1, on platelet-monocyte aggregation and on IL-1β synthesis. Both anti–P selectin and anti–PSGL-1 inhibited the aggregation of platelets with monocytes, as revealed by CD42b staining on CD14^+^ cells (Figure 5A). Antibodies against both P selectin and CD40L inhibited the synthesis of IL-1β by such monocytes, in particular anti–P selectin (Figure 5B). However, recombinant P selectin and CD40L could not directly induce the synthesis of IL-1β in monocytes (Figure 5C). These results indicated that platelet-monocyte aggregation is a prerequisite for platelet-dependent spike protein–induced monocyte activation through the engagement of P selectin/PGSL-1 and CD40L/CD40.

## Discussion

Platelets play a key role in the initiation of hemostasis and coagulation (6). Platelet activation has been reported in patients with COVID-19, and increased platelet activation was associated with disease severity (22). Exposure to plasma from patients with severe COVID-19 increased the activation of control platelets ex vivo (3). However, how platelets are activated in SARS-CoV-2 infection remains unclear. Platelets exhibit numerous surface receptors that mediate binding and entry of various viruses (23, 24) and their interaction with pathogens occurs through membrane receptors, including TLR, Fc receptors, and DC-SIGN. In the present study, we found that SARS-CoV-2 induced platelet activation by spike protein (Figure 1), and spike protein induced platelet activation through the engagement with CD42b (Figure 2). The biological relevance of these observations was underlined since we derived the same outcomes whether spike was present as a soluble protein or as part of a virus envelope. These findings confirmed that SARS-CoV-2 can activate platelets directly and identified CD42b as the binding receptor, although the binding affinity was only moderate (Supplemental Figure 3), leading to the delayed and modest activation response (Figure 1 and Supplemental Figure 2), while ACE-2 was not involved. Furthermore, ACE-2 was not detected as mRNA or protein in platelets (4, 5). Consistent with this, our results showed that ACE-2 was neither expressed on platelets (Supplemental Figure 4) nor recognized by SARS-CoV-2 during the interaction with platelets. Surprisingly, mRNA from the SARS-CoV-2 N1 gene was detected in platelets from patients with COVID-19, suggesting platelets may take up SARS-CoV-2 mRNA independent of ACE-2 (4). Although a study showed that platelets expressed both ACE-2 and TMPRSS2, a serine protease involved in spike protein priming (25), this apparently reverse result might be due to differences in methods to isolate platelets for RNA-based assays and in antibody binding epitopes and/or nonspecific binding for protein-based assays (26). CD42b, known as platelet glycoprotein Ib α chain (GPIbα), is a type I transmembrane protein identified as the ligand-binding portion of the GPIb-IX-V complex. The GPIb complex is best known as a major platelet receptor for vWF, which is essential for platelet adhesion and activation (27). Our results showed that spike protein competitively antagonizes vWF binding to GPIbα (CD42b) and interferes with platelet adherence to vWF (Figure 2F and Supplemental Figure 5, A and B). Although spike protein binding with CD42b might promote both prothrombotic and antithrombotic effects, it appears the prothrombotic mechanisms predominate if these processes are both active in patients infected with SARS-CoV-2 given that bleeding has not been reported clinically, but patients with severe COVID-19 frequently experience thrombosis and thromboembolism. To the best of our knowledge, this is the first description of CD42b as a SARS-CoV-2 spike protein binding receptor involved in platelet activation, although the question of whether SARS-CoV-2 infects platelets or megakaryocytes through CD42b needs to be investigated. CD42b may not be the only receptor by which spike protein interacts with platelets. While our data show this it is important, other possible receptors include PF4, CD147 (14), and CD26 (28).

For clarifying the intracellular signaling events during human platelet activation via the engagement of SARS-CoV-2 spike protein and CD42b, we examined the essential signaling molecules involved in the GPIb-IX-V complex and found that the engagement of spike protein with CD42b increased CD40L expression on platelets via PI3K/PKC signaling (Figure 3), which is involved in inside-out signaling in platelets (29). However, PI3K/PKC signaling was not involved in SARS-CoV-2 spike protein–induced P selectin expression (Figure 3D). Understanding the platelet signaling machinery is necessary to identify potential targets for the development of new agents that target platelets as a potential COVID-19 therapy.

Platelets not only actively participate in hemostasis but also play an important role in the immune response (6). Upon activation, platelets degranulate and express a repertoire of membrane receptors that enable them to bind to circulating leukocytes via P selectin (30). Platelets from patients with severe COVID-19 were able to induce tissue factor expression ex vivo in monocytes from healthy volunteers, suggesting a platelet-induced procoagulant activity through platelet-monocyte aggregation (3). In the present study, we found that SARS-CoV-2 spike protein did not induce monocyte activation directly, but SARS-CoV-2–activated platelets induced monocyte differentiation toward a proinflammatory phenotype, which featured higher expression of CD86, HLA-DR, and IL-1β (Figure 4). Similarly, platelet-monocyte aggregation is crucial in systemic inflammatory responses induced by influenza A (H1N1) (31), HIV, and Dengue virus (24). The presence of platelets skewed monocytes toward the type 1 macrophage phenotype in a cell contact–dependent manner via the GPIb/CD11b axis. Accordingly, platelet-licensed macrophages showed increased TNF-α levels and bacterial phagocytic activity and a reduced healing capability (32).

We further found that SARS-CoV-2–activated platelets mediated inflammatory monocyte activation through the engagement of P selectin/PGSL-1 and CD40L/CD40 in a cell contact–dependent manner (Figure 5). P selectin–mediated interactions in turn activate leukocyte signal transduction pathways (33), initiating the rapid formation of platelet-leukocyte aggregates (34). PSGL-1 is a leukocyte adhesion molecule involved in cell tethering and rolling on activated endothelium. PSGL-1 engagement induces tyrosine phosphorylation of Syk and SRE-dependent transcriptional activity in leukocytes (35). The ligation of CD40 on human monocytes induces increased expression of cell-surface proteins, including CD54, MHC class II, CD86, and CD40, and stimulates monocytes to produce TNF-α, IL-1β, IL-6, and IL-8 (36). Consistent with our data, although activated platelets express both soluble and membrane-bound CD40L (37), only the membrane-bound form (mCD40L) induces expression of proinflammatory cytokines and adhesion molecules on endothelial cells (38).

**Figure 6:**
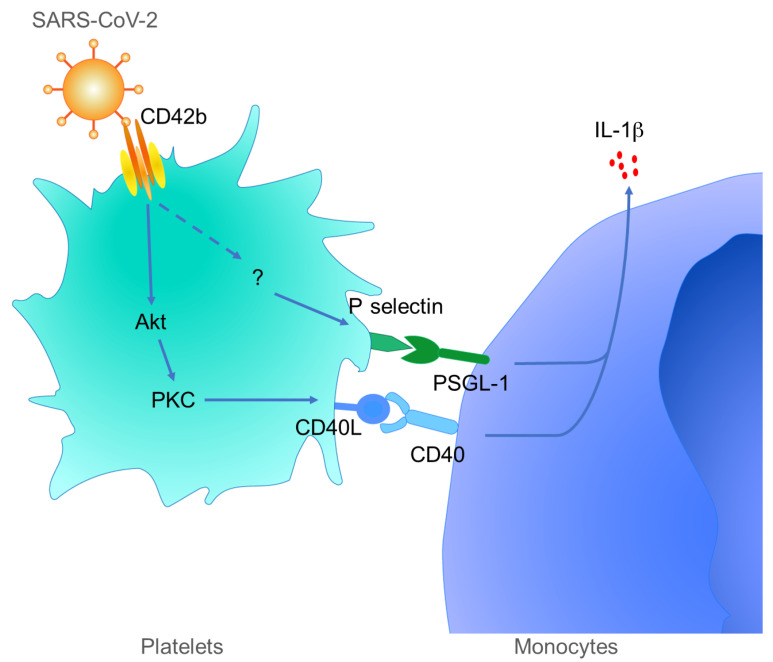
Schematic outline of platelet-mediated inflammatory monocyte activation by SARS-CoV-2 spike protein. SARS-CoV-2 spike protein interacts with CD42b to activate platelets, which express CD40L via PI3K/PKC signaling and P selectin via an unknown signaling pathway. The activated platelets induce inflammatory monocyte differentiation through the engagement of P selectin/PGSL-1 and CD40L/CD40.

In the present study, our data led us to propose a model of SARS-CoV-2 binding to platelets that explains the hypercoagulation, monocyte activation, and cytokine storm in severe COVID-19 (Figure 6). The SARS-CoV-2 virion binds to blood platelets using its spike protein, but binding is via CD42b rather than the ACE-2 receptor used to gain entry to many cell types (17). This results in the activation of multiple signaling pathways in the platelets, including an AKT-PKC cascade that upregulates CD40L expression and a distinct and so far unidentified pathway that upregulates P selectin. Both of these molecules bind to their known ligands on monocytes, PSGL-1 and CD40, resulting in monocyte activation, CD86 and HLA-DR expression, and IL-1β synthesis. We conclude that hypercoagulation and the cytokine storm in severe COVID-19 are linked through the spike-CD42b interaction that activates platelets, the CD40L-CD40 and the P selectin–PSGL-1 interactions that bind them to monocytes, and the strong induction of IL-1β in the monocytes. This mechanism may be a potential therapeutic target in severe COVID-19.

## Methods

### Spike protein and pseudotyped virus of SARS-CoV-2.

Recombinant spike protein (His tag) of SARS-CoV-2 was purchased from Sino Biological. Calcium phosphate–mediated transfection of HEK293T cells was used to generate pseudotyped virus as described previously (39), and the luciferase reporter HIV-1 proviral plasmid pNL-Δenv-luc and the expression plasmid pcDNA3.1-SARS-CoV-2-S (encoding for SARS-CoV-2 spike protein) were used for cotransfection. pcDNA3.1-SARS-CoV-2-S was a gift from Lu Lu (Fudan University, Shanghai, China). Pseudotyped particles released in the supernatant were harvested at 72 hours after transfection, mixed with PEG overnight, passed through a 0.45 μm SFCA low-protein binding filter, centrifuged at 500*g* for 5 minutes, aliquoted, and frozen at −80°C. Plaque formation was used for viral titration, and pseudovirus titers were determined to identify the TCID_50_; 100 μL viral stock was equivalent to 200 TCID_50_.

### Platelet and monocyte isolation.

Peripheral blood from healthy donors was drawn into sodium citrate by using 21G needles before centrifugation at 200*g* for 10 minutes at room temperature to obtain platelet-rich plasma. Platelet purification was performed as previously described (40, 41). In brief, platelets were washed by centrifugation at 1200*g* after a gel filtration using Sepharose 2B equilibrated with HEPES-buffered modified Tyrode’s buffer (10 mM HEPES, 150 mM NaCl, 2.5 mM KCl, 0.3 mM NaH_2_PO_4_, 12 mM NaHCO_3_, 0.2% BSA, 0.1% glucose, 2 mM EDTA, 100 nM PGE1). After magnetic removal of leukocytes by using CD45 microbeads (Miltenyi Biotec), platelets were resuspended to the concentration of 10^9^/mL in HEPES buffer, saline, or RPMI 1640 when indicated. Leukocyte contaminants after purification are shown as Supplemental Figure 1.

PBMCs were isolated from platelet-rich plasma–depleted blood samples by gradient centrifugation using Ficoll-Paque PLUS (GE Healthcare). Purified PBMCs were suspended in autoMACS Running buffer (Miltenyi Biotec), and monocytes were purified by using CD14 microbeads (Miltenyi Biotec) as previously described (42).

### Platelet stimulation and flow cytometry.

Platelet aggregation was measured by a flow cytometry–based assay (43). Briefly, purified platelets were labeled with either CFSE (0.15 μM, Thermo Fisher Scientific) or PKH26 (2 μM, Sigma-Aldrich) and mixed 1:1 supplemented with 0.1× volume of autologous platelet-poor plasma. Samples were incubated with or without spike protein or S-pseudovirus for 15 minutes, fixed by adding 5× volume of 1% formaldehyde, and detected by FACSCanto (BD Biosciences). All populations were gated to identify platelets, and the percentage of double-colored events indicates the extent of aggregation.

Platelet-monocyte aggregation experiments were performed as previously described (41). In brief, diluted whole blood was incubated with serial dilutions of spike protein or S-pseudovirus in the presence of APC-conjugated anti-CD42b (BD Pharmingen, 551061) and V450-conjugated anti-CD14 (BD Horizon, 561390) for 10 minutes. For blocking experiments, anti–P selectin (10 μg/mL, R&D Systems, BBA30) and anti–PSGL-1 (10 μg/mL, R&D Systems, MAB3345) were added for 5 minutes before spike protein and S-pseudovirus as indicated in the experiments. After fixation and lysing erythrocytes by using FACS lysing solution (BD Biosciences), samples were analyzed by FACSCanto or Gallios (Beckman Coulter).

Purified platelets were incubated with recombinant spike protein or S-pseudovirus in the presence of PE-conjugated anti–P selectin (BioLegend, 550561; or in some experiments, APC-conjugated anti–P selectin, 304910), Pacific blue–conjugated anti-CD40L (BioLegend, 310820), AF488-conjugated anti-His tag (R&D Systems, IC0501G, for spike protein detection), AF647-conjugated anti–ACE-2 (BioLegend, 375803), and APC-conjugated anti-CD42b (BD Pharmingen, 551061; or PE-conjugated anti-CD42b in indicated experiments, 555473) for 10 minutes as previously described (22). After fixation by 1% formalin, platelets were analyzed by flow cytometry. In blockade experiments, anti-spike RBD (10 μg/mL, Sino Biological, 40592-R001), anti-CD42b (20 μg/mL, eBioscience, 14-0429-82), isotype antibody (R&D Systems, MAB005), Ro 31-8220 (2 μM, MedChemExpress), and LY294002 (10 μM, Sigma-Aldrich) were added to platelets before stimulation and staining.

### Fluorescence imaging.

Platelet-monocyte aggregation experiments were performed as above. After fixation and lysing erythrocytes, samples were immobilized on microscope slides by using Cytospin (Cytopro, ELITech) (44) and visualized by fluorescence microscopy (Olympus IX51).

### IP and Western blot.

Purified platelets were incubated with recombinant spike protein or S-pseudovirus for 5 minutes before washing and lysis in RIPA buffer. To precipitate spike protein, samples were incubated with Sepharose bead–conjugated anti-His (Cell Signaling Technology) overnight at 4°C and washed using RIPA buffer according to the manufacturer’s protocol. Denatured protein samples were blotted by using anti-ACE-2 (R&D Systems, AF933), anti-GRP78 (Novus Biological, NB100-56411), anti-TLR2 (R&D Systems, AF2616), anti-CD42b (Abcam, EPR19204), anti–S-RBD (Sino Biological, 40592-R001), anti–phospho-(Ser) PKC substrates (Cell Signaling Technology, 2261), anti–phospho-(Ser473) Akt (Cell Signaling Technology, 4060), and anti–β-actin (Cell Signaling Technology, 3700) after electrophoresis using SDS-PAGE. Proteins were transferred to a PVDF membrane as previously described (42). Results were analyzed by Image Lab (Bio-Rad).

The direct binding assays were carried out using 1 μg/mL recombinant S-RBD (mFC tag, Sino Biological), CD42b (His tag, Sino Biological), and ACE-2 (His tag, Sino Biological). Mixed proteins were incubated and precipitated overnight by Sepharose bead–conjugated anti-His (Cell Signaling Technology, 4079) or Protein G Agarose beads (Cell Signaling Technology).

In blockade experiments, anti-CD42b (20 μg/mL, eBioscience, 14-0429-82), anti–ACE-2 (20 μg/mL, R&D Systems, AF933), anti-GRP78 (10 μg/mL, Novus Biological, NB100-56411), anti-TLR2 (10 μg/mL, R&D Systems, MAB2616), GPVI Fc chimera protein (R&D Systems, 10452-GP), isotype antibody (R&D Systems, MAB005), Ro 31-8220 (2 μM, MedChemExpress), LY294002 (10 μM, Sigma-Aldrich), and Benztropine mesylate (25 μM, MedChemExpress) were added to platelets for 5 minutes before incubation with spike protein.

### Flow cytometry for vWF binding.

Fixed platelets were incubated for 30 minutes with recombinant vWF (His tag, Sino Biological) and ristocetin (Chrono-log) at the indicated concentrations as described (45). S-RBD was added to platelets 15 minutes earlier than vWF. AF488-conjugated anti-His (R&D Systems, IC0501G) was used to detect the platelet-vWF complexes.

### Platelet and monocyte cocultures.

Purified monocytes and autologous platelets were cocultured with recombinant spike protein or S-pseudovirus in RPMI 1640 medium for 10 minutes. Monocytes were washed by centrifugation at 200*g* to remove spike protein, S-pseudovirus, and untethered platelets before culturing in RPMI 1640 medium supplied with 10% FBS for 12 hours (for intracellular staining) or 48 hours (for phenotype measurements). In blockade experiments, anti–P selectin (10 μg/mL, R&D Systems, BBA30) and anti-CD40L (10 μg/mL, R&D Systems, MAB3345) were added before spike protein and S-pseudovirus in indicated experiments.

### Phenotype and intracellular cytokine staining of monocytes.

Cell surface and intracellular cytokine staining of monocytes was performed as previously described (46). Briefly, cultured monocytes or purified monocytes were washed in PBS and stained by PE-conjugated anti-CD80 (BD Pharmingen, 557227), FITC-conjugated anti-CD86 (BD Pharmingen, 555657), PE-conjugated anti-CD163 (BD Pharmingen, 556018), FITC-conjugated CD206 (BD Pharmingen, 551135), PE-Cy7–conjugated HLA-DR (BD Pharmingen, 560651), or APC-conjugated anti-CD42b (BD Pharmingen, 551061) before analysis by FACSCanto. Fixation and permeabilization were performed using Cytofix/Cytoperm Plus kit with GolgiPlug (BD Biosciences) and stained by PE-conjugated anti–IL-1β (R&D Systems, IC201P) and PE-CF594–conjugated anti–IL-10 (BD Horizon, 562400) before analysis. All data acquired from flow cytometry were analyzed with FlowJo (Treestar software).

### Statistics.

All statistical tests were performed using GraphPad Prism. For the in vitro experiments, a 2-tailed paired Student’s *t* test was used to compare differences between 2 paired groups; a 1-way ANOVA with Dunnett’s multiple-comparison test was used to compare more than 2 matched groups (Figure 2B and Figure 3, B and D). All groups were compared with each other unless otherwise noted here or in the figure legends. Data represent mean ± SD. *P* values of less than 0.05 were considered statistically significant.

### Study approval.

All studies were conducted according to the experimental practices and standards that were approved by the Medical Ethics Committee of The First Hospital of Jilin University (approval code 2021-297).

## Author contributions

ZT, GL, and TL conceived and designed the study. TL, YY, YL, ZW, FM, and GZ acquired and analyzed data. RL, XX, JW, JN, GL, and INC contributed to experimental design and interpretation of the results. ZT, GL, INC, and TL wrote and revised the manuscript.

## Supplementary Material

Supplemental data

## Version 1 12/29/2021

In-Press Preview

## Version 2 02/15/2022

Electronic publication
