# Supplementary material for: Platelets mediate inflammatory monocyte activation by SARS-CoV-2 spike protein
Source: J Clin Invest. 2022 Feb 15;132(4):e150101. doi: 10.1172/JCI150101 (PMC8843740; doi:10.1172/JCI150101)
Supplement: Supplemental data [file jci-132-150101-s064.pdf]

Supplemental Materials for

Title: Platelets mediate inflammatory monocyte activation by SARS-CoV-2 Spike protein

Authors:

Tianyang Li[1], Yang Yang[1], Yongqi Li[1], Zhengmin Wang[1], Faxiang Ma[1], Runqi Luo[2], Xiaoming Xu[2], Guo Zhou[2], Jianhua Wang[3], Junqi Niu[4], Guoyue Lv[4*], Ian Nicholas Crispe[5], Zhengkun Tu[1,4*].

Affiliations:

[1] Institute of Translational Medicine, The First Hospital of Jilin University, Changchun, Jilin, China.

[2] Infectious Disease, The Second Affiliated Hospital of Guangzhou Medical University, Guangzhou, Guangdong, China.

[3] Guangzhou Institutes of Biomedicine and Health, Chinese Academy of Sciences, Guangzhou, Guangdong, China.

[4] Institute of Liver Diseases, The First Hospital of Jilin University, Changchun, Jilin, China.

[5] Department of Laboratory Medicine and Pathology, University of Washington, Seattle, WA, USA

***Correspondence to:** Zhengkun Tu, MD. Email: tuzhengkun@jlu.edu.cn;

Guoyue Lv, MD, PhD. Email: lvgy@jlu.edu.cn.

This PDF file includes:

Supplemental Figure 1-8

Supplemental Methods

Cell line

Huh7 cells which endogenously express ACE-2 (1) were maintained in Dulbecco's modified Eagle's medium (DMEM) containing 10% heat-inactivated fetal bovine serum (FBS) before flow cytometry, as described in Methods.

Platelet aggregation assay

Platelet aggregations were measured on an optical aggregometer (AG800, Techlink, Beijing) at 37°C as described (2). Briefly, 300 μl of PRP was stimulated by S protein (1 μg/mL) or S-pseudovirus (80 $TCID_{50}$/mL) and light transmission was recorded until trace stabilization.

Biolayer interferometry

Biolayer interferometry analysis was performed on an Octet RED96 (ForteBio) as described (3). Spike RBD protein (20 μg/mL) were immobilized on AMC sensors to maximum loading levels. Associations and dissociations were performed in PBS supplemented with 0.1% BSA, 0.02% Tween20 at 30°C after sensors were washed to a obtained baseline. K_D value was analyzed using DataAnalysis9 (ForteBio).

Platelet-derived dense granule secretion assay

Platelet ATP/ADP secretion from dense granules was detected as described (4). Briefly, 80 μl of PRP was incubated with or without S protein and collagen at indicated concentrations for 5 min at room temperature on an orbital shaker, and 10 μl of Chrono-

lume (Chrono-Log) were injected into each well. Luminescence was detected by Synergy H1 (Biotek).

Enzyme-linked immunosorbent assay (ELISA)

The monocyte culture supernatants were collected, and the concentration of IL-1β, TNF-α and IL-10 were measured by ELISA Ready-Set-Go® (eBioscience, San Diego, USA) according to the manufacturer's instructions (5).

Supplemental Figures

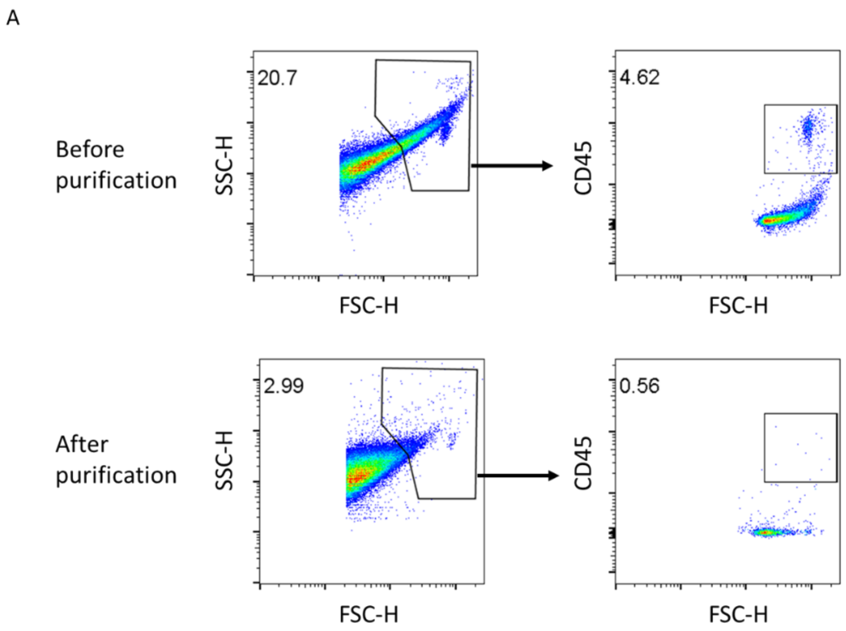

Supplemental Figure 1: Leukocyte contaminants in purified platelets.

(**A**) Before and after purification, platelets were stained by anti-CD45 and analyzed by flow cytometry, and leukocyte contaminants were shown.

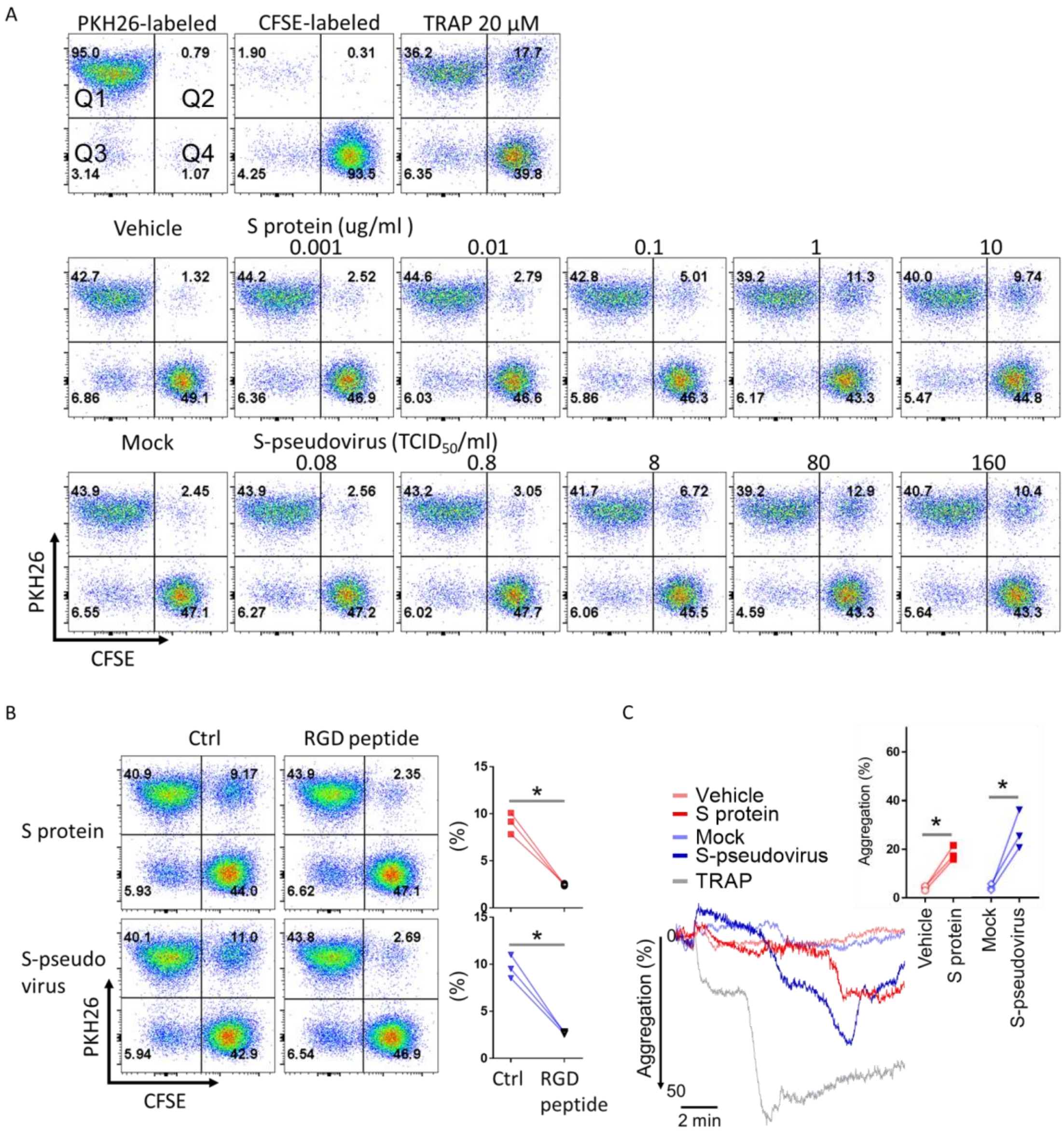

Supplemental Figure 2: SARS-CoV-2 induced platelet aggregation.

(**A**) Purified platelets were labeled by PKH26 or CFSE and mixed 1:1 before incubation with S protein or S-pseudovirus at indicated concentration. Platelets stained with PKH26 are enclosed in Quadrant 1. Platelets stained with CFSE are enclosed in Quadrant 4. Double-colored events in Quadrant 2 represent platelet aggregates before and after stimulation. Statistical analysis was displayed in Figure 1A. (**B**) Purified platelets were labeled by PKH26 or CFSE and mixed 1:1 before incubation with S protein (1.0 μg/mL) or S-pseudovirus (80 TCID50/mL) in the presence or absence of

RGD peptide. (**C**) Representation and quantification of light transmission curve of platelets aggregation stimulated by S protein or S-pseudovirus were shown. Mean with SD and *P* value by paired Student's *t* test are displayed. * Indicated P value < 0.05.

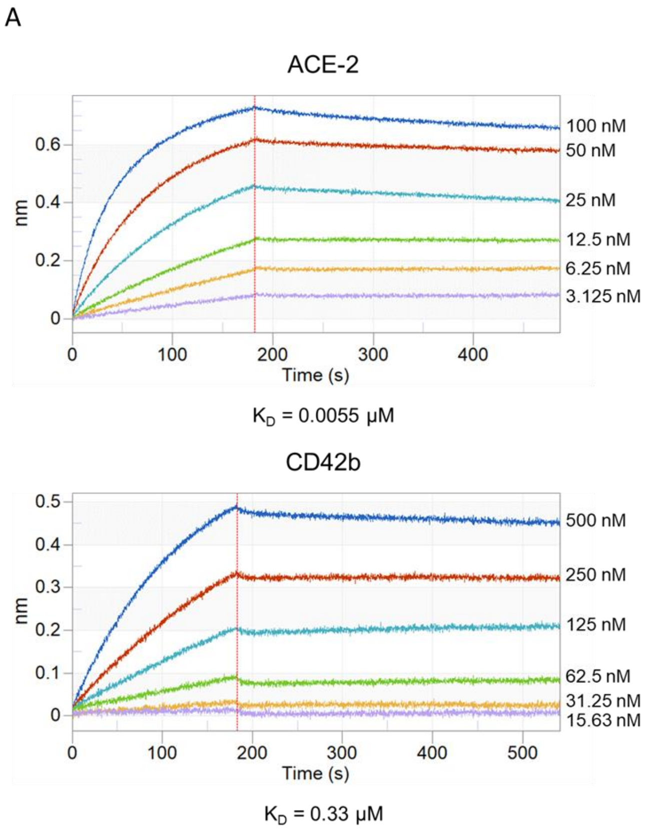

Supplemental Figure 3: The binding affinity of recombinant Spike protein to recombinant CD42b and ACE2 were characterized by kinetics.

(**A**) Binding curves of immobilized Spike RBD with the ACE-2 (upper) and CD42b (lower) are shown. K_D are analyzed by fitting the data to a 1:1 binding model.

A

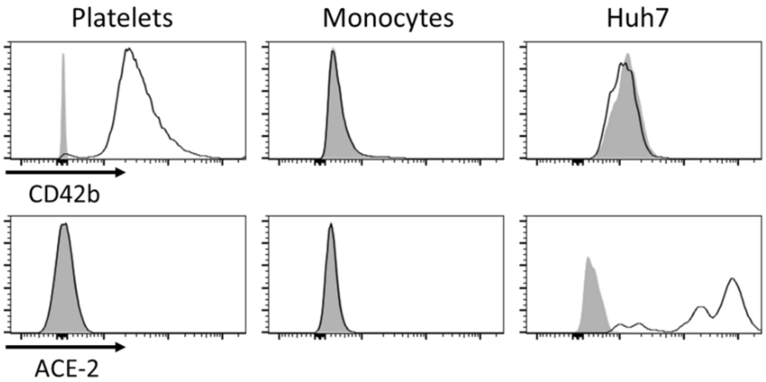

Supplemental Figure 4: ACE-2 was not detected on platelets.

(**A**) ACE-2 and CD42b expression on purified platelets, monocytes and the Huh7 cell line (which endogenously expresses ACE-2) was tested by flow cytometry.

A

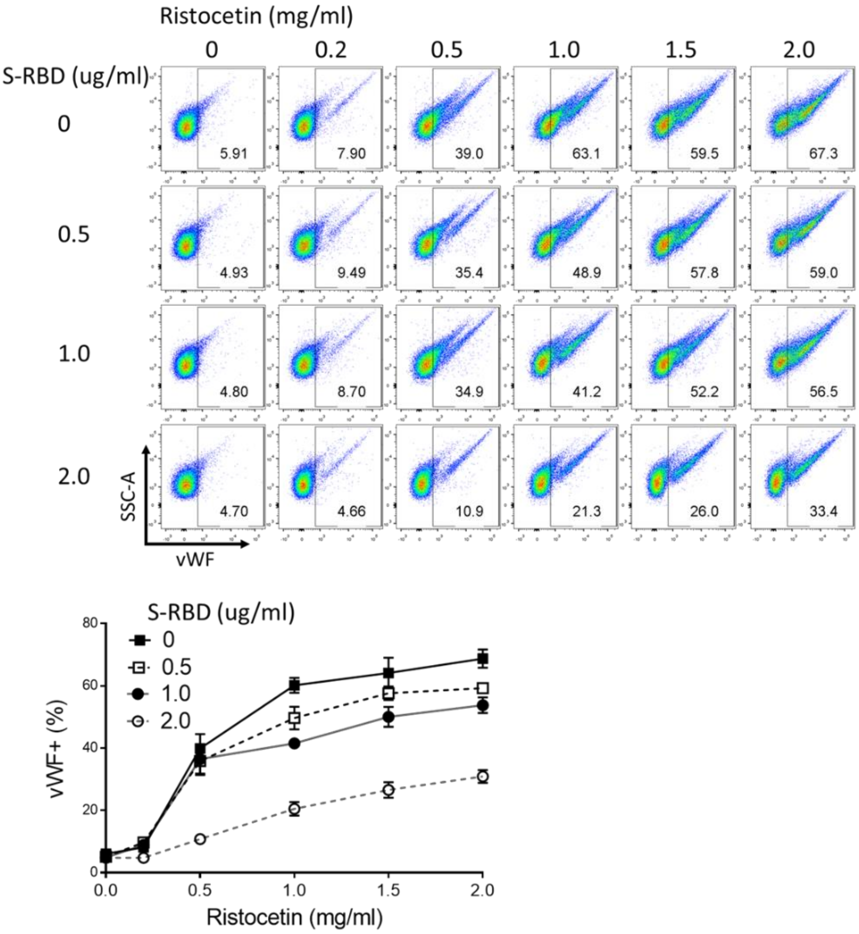

B

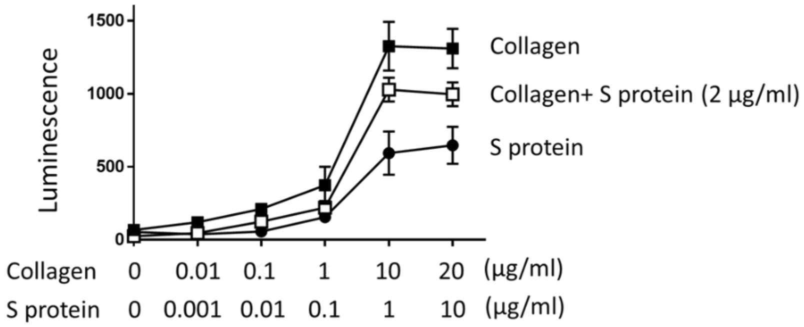

Supplemental Figure 5: Spike protein impeded vWF binding on platelets.

(**A**) Platelet-vWF complexes were detected by flow cytometry using recombinant vWF and ristocetin. MFI analysis was shown in Figure 2F. (**B**) Platelet-rich plasma was incubated with S protein and collagen at indicated concentrations, and luminescence derived from ATP/ADP release were measured. Data are reported as the mean with SD.

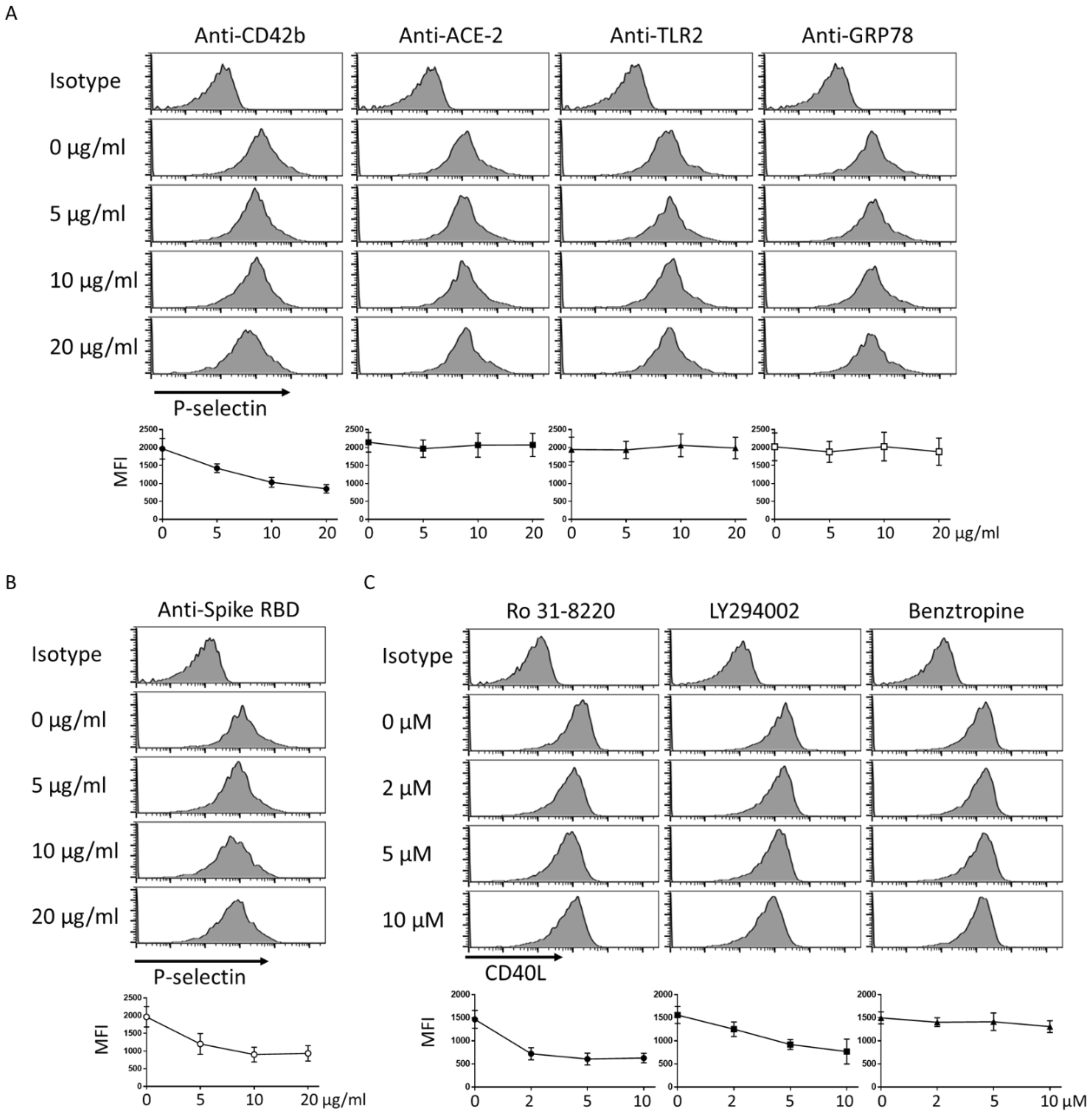

Supplemental Figure 6: Dose response curves for inhibitory antibodies and inhibitors.

Purified platelets were incubated with indicated inhibitory antibodies before stimulation with 1 µg/ml of S protein (**A**, **C**) or with 80 $TCID_{50}$/mL of S-pseudovirus (**B**). P-selectin or CD40L expression was detected by flow cytometry. Data are reported as the mean with SD.

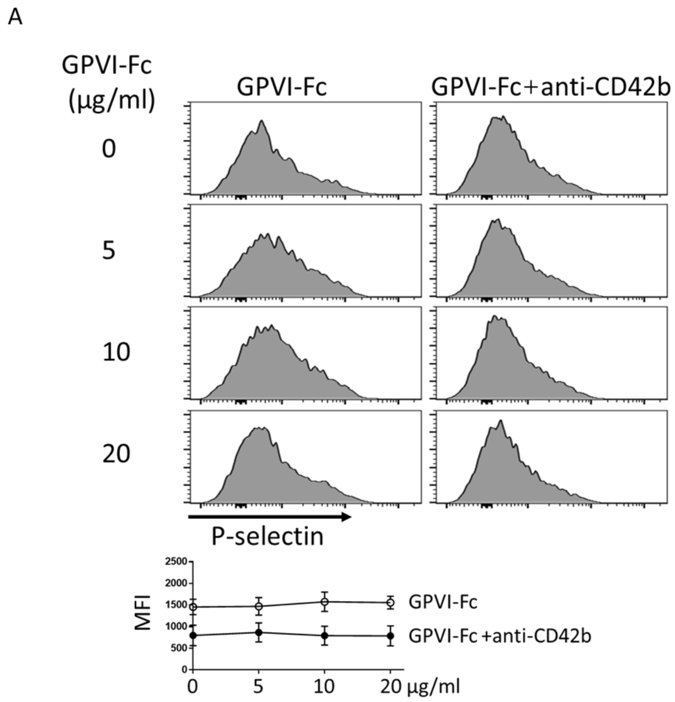

Supplemental Figure 7: Blockade of GPVI did not affect Spike protein binding with CD42b-induced P-selectin expression.

(**A**) Purified platelets were incubated with S protein (1 μg/ml) and GPVI-Fc chimera protein at indicated concentrations in absence or presence of anti-CD42b (20 μg/ml). P-selectin expression was detected by flow cytometry. Data are reported as the mean with SD.

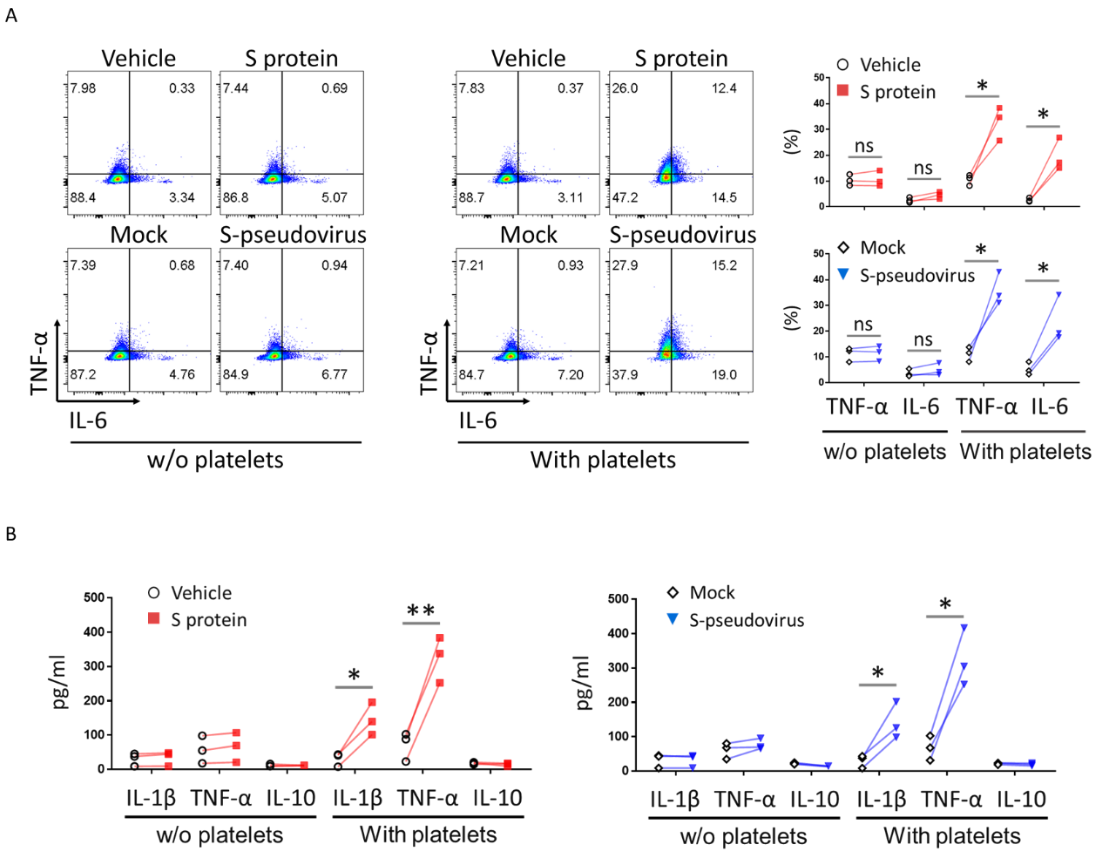

Supplemental Figure 8: SARS-COV-2-activated platelets mediated IL-6 and TNF-α expression in monocytes.

(**A**) Purified monocytes (n=3) were cultured and co-cultured as described in Figure 4B, and IL-6 and TNF-α expression is shown. (**B**) IL-1β, TNF-α and IL-10 levels in supernatants from monocyte culture were measured by ELISA. *P* value by paired Student's *t* test is displayed. * indicated *P* value < 0.05, ** *P* < 0.01, *ns* indicated not significant.

Supplemental references

1. Lai ZW, et al. The identification of a calmodulin-binding domain within the cytoplasmic tail of angiotensin-converting enzyme-2. *Endocrinology.* 2009;150(5):2376-81.
2. Dai B, et al. Integrin-αIIbβ3-mediated outside-in signalling activates a negative feedback pathway to suppress platelet activation. *Thromb Haemost.* 2016;116(5):918-30.
3. Tortorici MA, et al. Structural basis for human coronavirus attachment to sialic acid receptors. *Nat Struct Mol Biol.* 2019;26(6):481-9.
4. Sun B, et al. Luminometric assay of platelet activation in 96-well microplate. *Biotechniques.* 2001;31(5):1174, 6, 8 passim.
5. Li H, et al. Regulatory NK cells mediated between immunosuppressive monocytes and dysfunctional T cells in chronic HBV infection. *Gut.* 2018;67(11):2035-44.
